# Regaining momentum for international climate policy beyond Copenhagen

**DOI:** 10.1186/1750-0680-5-2

**Published:** 2010-06-04

**Authors:** Michael Huettner, Annette Freibauer, Constanze Haug, Uwe Cantner

**Affiliations:** 1 Max-Planck-Institute for Biogeochemistry, Hans-Knoll-Str. 10, 07745 Jena, Germany; 2 GSBC-EIC 'The Economics of Innovative Change', Friedrich-Schiller-University Jena, Carl-Zeiss-Str. 3, 07743 Jena, Germany; 3 Johann Heinrich von Thünen-Institute, Institute of Agricultural Climate Research, Bundesallee 50, 38116 Braunschweig, Germany; 4 Institute for Environmental Studies, Vrije Universiteit Amsterdam, De Boelelaan 1087, 1081 HV Amsterdam, The Netherlands

## Abstract

The 'Copenhagen Accord' fails to deliver the political framework for a fair, ambitious and legally-binding international climate agreement beyond 2012. The current climate policy regime dynamics are insufficient to reflect the realities of topical complexity, actor coalitions, as well as financial, legal and institutional challenges in the light of extreme time constraints to avoid 'dangerous' climate change of more than 2°C. In this paper we analyze these stumbling blocks for international climate policy and discuss alternatives in order to regain momentum for future negotiations.

## Introduction

The last 20 years have witnessed increasing momentum for international environmental policy efforts in order to avoid 'dangerous' anthropogenic climate change. Major achievements in the process so far have been the adoption of the United Nations Framework Convention on Climate Change (UNFCCC) in 1992 and the Kyoto Protocol (KP) in 1997, which entered into force in 2005. From 2005 onwards, Parties increasingly focused on the negotiations of the global climate regime beyond 2012. The 2007 Bali Action Plan kicked off the actual negotiations on a post-2012 climate agreement, producing a comprehensive negotiations roadmap that was to lead the way to the adoption of a new treaty at the climate conference in Copenhagen in December 2009 [1]. After very limited progress at a series of meetings prior to Copenhagen, the initial ambitions for reaching a legally binding agreement in Copenhagen had been reduced to the hope of reaching political consensus on the key building blocks of the Bali Action Plan. However, the summit largely failed to deliver on this objective. Its main result, the 'Copenhagen Accord' (CA) which had been negotiated by a group of Parties led by the US and the largest emerging economies, did not gain the endorsement by the plenary of the Conference of the Parties (COP), and was merely 'taken note of'. All in all, the substantial advances made by Copenhagen put the world on a trajectory beyond 2°warming by the end of this century.

At the same time, combating climate change has become urgent. Global carbon dioxide emissions have risen by approximately 41% since 1990 [2]. If 'dangerous' climate change of more than 2°C is aimed to be prevented with 67% probability, then global emissions should peak between as early as 2015 and 2020. If the upper end of this deadline is chosen, annual global emission reductions have to amount to 9.0% from 2020 onwards. Since it remains questionable whether societies are technologically and politically capable of achieving such rapid reductions, the 2015 deadline requiring an annual emission reduction rate of 5.3%, while still very ambitious, is probably more realistic [3]. Consequently, participation in global mitigation efforts needs to be broadened, the ambition of reduction targets and policies strengthened, and necessary financing secured within the narrow timeframe of five to ten years.

Even if sufficient emission reductions targets were negotiated under a binding treaty in Mexico in December 2010, we still would have to be aware of the fact that agreeing on a climate treaty is not equivalent to achieving emission reductions. On the one hand, the time between the negotiation of a treaty and its entry into force can span several years as the experience of the KP taught us. On the other hand, the translation of an international climate treaty into national law and finally into effective national and local emission reduction measures might also take several years. COP decisions could be translated into domestic actions much faster than treaties, but they have weaker legal status risking the domestic implementation. Research findings on the implementation of the KP also reveal that climate mechanisms such as the Clean Development Mechanism (CDM) might only work imperfectly and could need lengthy adjustment measures [4].

International climate negotiations so far have been based on the notion that a global challenge requires a global solution, that the United Nations are the most suitable platform for universal action, and that with time it will be possible to increase the ambition level and stringency of the commitments, drawing in a growing number of Parties [5]. This reflects the commonly held assumption that multilateral environmental agreements typically gradually evolve from loose political statements towards an institutional framing of the problem and finally into binding, enforceable agreements (e.g. [6]). This is achieved by iteratively bringing a large amount of actors together to steadily increase their commitment. Major breakthroughs in such a process are often engineered in arduous all-night negotiations, with the psychology of political pressure as a key factor (e.g. [7]). Up to Copenhagen, the climate regime seemed a typical example of this logic. However, the Copenhagen negotiations have demonstrated the institutional, structural and strategic limitations of the approach, when applied to a topic as complex as climate change. In this paper, we first examine key characteristics and controversies in international climate policy that help put the outcome of Copenhagen into context. In a second step, we then point to some options and alternative approaches for regaining momentum under the international climate regime.

## Stumbling blocks in the climate negotiations to Copenhagen

### Unfavorable institutional settings

United Nations processes, including the climate negotiations, often follow the consensus principle. This ensures maximum legitimacy and credibility among Parties. Yet reaching agreement among 192 of them with fundamentally different visions of the problem, its consequences and the ways of resolving it severely limits prospects for progress. Any outcome needs to take into account the concerns even of the most unwilling country since it cannot be overruled as in a major voting context. Consequently, the UN procedures inherently tend to incentivize extreme positions, slowing down the negotiation progress, while proactive forerunners tend to be disincentivized by lack of recognition. In Copenhagen the consensus principle for COP decisions was (ab)-used various times to suspend the COP for strategic purposes. Furthermore, several countries took advantage of the system in order to bargain for national interests in exchange for their approval of the final decision. From a structural perspective it is thus questionable if the current format of the UNFCCC conference is an appropriate venue for negotiating meaningful climate agreements. With a view to overcoming all these difficulties, during the opening session of COP15, one party suggested the alternative of two-thirds majority voting [8]. While this might theoretically be a sensible approach, it is practically unfeasible to attain since the voting procedure under the UNFCCC can only be changed by a consensus vote.

The way in which the Copenhagen Accord was eventually negotiated effectively shunned the elaborate UN rules and procedures for decision-making. The twelve-paragraph-long text, which hardly built on the two years of arduous drafting work by UNFCCC working groups, was the product of closed-door deliberations by a small group of heads of states at the margins of the negotiations, led by the US and the BASIC country group (Brazil, China, India and South Africa). Their announcement to the world public that a deal had been reached even preceded its full discussion in the COP plenary. Not surprisingly, very heated discussions ensued when the text was formally introduced to the plenary. Ultimately, due to the opposition of five countries, the Accord was not adopted, but merely "noted" by the COP. Overall, these events have created a general atmosphere of mistrust, which will complicate future negotiations [9,10].

### Climate change as a collective action problem

International climate policy is still trapped in a fundamental collective action dilemma. When labeling climate protection as a global public good, then inaction on climate measures can be viewed as international prisoners' dilemma. Climate policy can thus act as means of cooperation in order to overcome this public good problem. Still, countries might try not to cooperate by not signing an international ambitious climate agreement. It would allow them to share the benefits of climate protection, while not bearing the costs of associated measures. Here, non-cooperation does not only mean blocking such agreement but also proposing emission reduction targets (and financing provisions for developing countries), which remain below their "global responsibility" (see below). Disputes over non-cooperation due to insufficient emission limitation targets hampered the negotiations in Copenhagen - most prominently between the US and China. The US delegation was unwilling to increase their offered target of 17% emission reduction compared to 2005, since this would have threatened its later approval by the US senate. The announced US target itself was viewed insufficient by China, which in turn rejected binding and ambitious targets for its economy. The US senate and US congress in turn state that they will only approve more ambitious targets, if economies in transition such as China take up binding targets [11]. This vicious circle exists in various other constellations, whereas it remains speculative if mistrust or the aim to continue non-cooperation prevent consensus.

### Burden-sharing and a deepening North-South divide

The required levels of ambition under a new agreement would clearly have to move beyond the 5.2% emission reductions (compared to 1990) under the KP, which can only be viewed as 'test-run' of an environmentally effective climate agreement. However, there is no point-landing on a certain temperature target, but rather probabilities. When a political decision on the accepted probability has been made, then this provides information on the global allowable atmospheric greenhouse gas (GHG) concentration [3]. This concentration could then be translated into the allowable global emission budget. How this budget is finally divided among countries as individual emission reduction targets is a highly controversial political decision, since it implies major economic restructuring towards low-carbon societies [12].

The science also makes clear that the challenge of reducing emissions can no longer be viewed as the sole responsibility of industrialized countries. Besides the need for considerably more ambitious emission reduction targets and financing commitments by industrialized countries, deviations from the emission trajectories in emerging economies are a *sine qua non *for avoiding 'dangerous' climate change [13]. If there were any hopes that the political divide between the North and the South has narrowed since its first manifestation at the UN Conference on the Human Environment in 1972, the experience of Copenhagen provided evidence to the contrary [9]. The rift was not only visible in the vehement opposition of emerging economies to commit to binding emission targets. The self-confident negotiation stance of developing countries on such issues as technology transfer, adaptation financing and reducing emissions from deforestation, forest degradation and the enhancement of carbon stocks (REDD+) underscored their recognition of mutual dependence for reaching a post-2012 agreement. At the same time, the repercussions of the financial crisis hampered the willingness of industrialized countries to provide funding in the magnitude envisaged by developing countries.

At the most basic level, reaching agreement in Copenhagen was complicated by very different views on the notions of "equity" and "fairness" between North and South [14]. Particularly the United States and Australia stipulated that, based on their current share in global emissions and future growth trends, emerging economies should assume substantial commitments for cutting emissions beyond 2012. The North thus followed a forward-looking notion of equity and fairness, while developing countries reasoned from a largely historical perspective, calling for emissions in the past 200 years to be considered in distributing "allowed emission budgets".

### Procedural and substantial complexity

Copenhagen certainly proved one of the most procedurally and substantially complex negotiations in human history. Two years of work in numerous subgroups under two different negotiation tracks had resulted in hundreds of pages of heavily bracketed negotiation text on a large number of often interdependent issues. Boiling this down to a workable draft agreement proved an impossible challenge, both at the last meeting prior to Copenhagen and at the summit itself [15]. Overall, the complexity of the subject matter has clearly increased since the Kyoto conference. In addition to the standing items on the UNFCCC's agenda, a large number of new topics have entered the negotiations agenda. REDD+, adaptation and technology transfer financing, national appropriate mitigation actions (NAMAs), capacity building on measuring, reporting and verifying emission reductions as well as many other items needed to be integrated in the negotiations with multiple interlinkages and dependencies among the various agenda items. This setting included a much broader array of heterogeneous stakeholders, country circumstances and interests than ever before in the history of the UNFCCC. The complexities and interlinkages blocked partial progress on individual negotiation items and would only have been resolved in a final package decision. Experienced delegates thus described the latest climate negotiations as "too political for the technicians and too technical for the politicians" [16].

### Legal nature and architecture of a future agreement

The architecture and binding or non-binding nature of a future climate agreement and whether to pursue the path of the Kyoto Protocol, and to combine rules for industrial countries with those for developing countries or not were major issues of dissent in Copenhagen. The US position not to join the KP in a second commitment period was mainly related to concerns about remaining legal obligations under the KP and to domestic political constraints. The EU pushed strongly for a single protocol approach, in which the content of the 'Ad-hoc Working Group on Long-term Cooperative Action' (AWG-LCA) and the 'Ad Hoc Working Group on Further Commitments for Annex I Parties under the Kyoto Protocol' (AWG-KP) would merge towards one legally binding protocol. Most developing countries advocated non-binding regulations for Non-Annex-1 countries and a legally binding future KP. This would however bear the risk of endorsing double standards for climate protection by potentially subjecting Annex-1 countries to stricter rules under the KP [17]. Some developing countries, certainly the Alliance of Small Island States (AOSIS), but also South Africa and Brazil, supported a double protocol approach with a legally binding outcome in the AWG-LCA. Yet this was rejected by China and India since they feared moving towards a binding emission reduction agreement. Developing countries largely rejected the EU proposal, since it would abandon the existing Kyoto architecture and could lead to lengthy re-negotiations on institutional settings and rules [18].

### Sources and management of climate financing

Another critical building block of the Bali Action Plan addresses the financing of REDD+, adaptation, capacity building and technology transfer in developing countries. Industrial countries have generally accepted that they bear the historical responsibility for anthropogenic climate change, and therefore have to deliver financial support. However, the magnitude of these payments, the division of financing among donors, as well as the requirements for managing the financial transfers remains highly contentious. Between 2010 and 2050 the costs of adapting to an approximately 2°C warmer world by 2050 were estimated to range between USD 75 and 100 billion a year [19]. The CA's pledge to provide funds approaching USD 30 billion for the period 2010-2012, as well as mobilizing jointly USD 100 billion per year by 2020 is often perceived as the greatest success of COP 15. But these funds should not only cover the costs of adaptation, but also those of mitigation, technology transfer and capacity building actions [20]. Hence, even assuming that all funding under the CA would indeed be new and additional, it might only be sufficient to cover the impacts resulting from 1.5°C of warming [21]. Article eight of the CA states that "funding will come from a wide variety of sources, public and private, bilateral and multilateral, including alternative sources of finance" [20]. This could include tapping carbon markets through international emission trading for mitigation actions in developing countries. While carbon markets could indeed deliver a portion of the needed funding, their unclear link with emission reduction targets of donor countries after Copenhagen poses the risk of lowering their ambitions through offsetting (see e.g. [22]. The CA also established a new Copenhagen Green Climate Fund. However, how this instrument relates to the already existing funding mechanisms under the Convention and the Protocol and how it could be made operational given the lack of legal status of the CA, remains to be seen [23].

### Short-term incentives and notion of success

Whilst the CA demonstrates consensus about the urgency of mitigation and the long-term 2°C goal, the implications of this consensus at the level of decision making - regional to national, immediate to decadal - remain unclear. Without a clear vision about social welfare benefits of mitigation actions there is no short-term incentive to initiate mitigation actions. So far the scientific consensus that some change in climate is unavoidable and hence societal change to adapt to it is inevitable (see e.g. [24]) has not yet reached the spirit of the negotiations. As long as success in Copenhagen is interpreted by critical negotiators and interest groups as resistance to change a consensus based negotiation process is prone to fail to achieve the required momentum for adequate ambition and timing of mitigation actions.

The CA itself did neither provide medium-term (2020) nor long-term (2050) emission reduction targets. Countries were however encouraged to submit their pledges on reducing their own GHG emissions until 2020 by 31st January 2010. These pledges translate into emission reductions of 11-19% (below 1990, excluding forestry and land use change emissions) for industrialized countries and roughly 5% -16% (below business as usual) for emerging economies and developing countries [25]. The determination of the latter emission reduction range should however only be viewed as tentative, since the voluntary targets of the major economies in transition China and India refer to emission intensity targets. These pledges would lead to approximately 2.9-3.9°C of temperature increase by 2100 [26], which does not only contradict the targeted temperature limit in the CA, but also threaten the overall aim of the UNFCCC.

## Ways forward

We hope to have shown that the current approach to international climate policy looks insufficient to reach a fair, ambitious and binding agreement and broad participation in the short time frame available. While some hope that the upcoming climate conferences in Mexico and in South Africa will eventually produce the long awaited, ambitious post-2012 climate treaty, other experts argue that the CA might well represent the high-water mark of the climate change regime for some time to come [18]. However, the urgency to increase efforts in emissions abatement and adaptation financing prohibit a further business-as-usual policy for the climate regime.

Having outlined some key factors that have obstructed progress, in the following we present several suggestions how to overcome the stumbling blocks for a post-2012 agreement in the light of the complex requirements and conditions. We concentrate on the mentioned structural shortcomings of negotiations under the UNFCCC as well as the inability to adequately address the highly complex negotiation agenda, taking account of the large number of Parties involved. We also provide suggestions on how to resolve issues of prevailing mistrust among Parties and strategic bargaining on financing and mitigation burden sharing in the light of the perceived fundamental collective action dilemma.

### Building institutional complementary processes

The UNFCCC process alone will likely not be sufficient to achieve swift progress on the long list of unresolved, highly controversial negotiation issues. We do not argue for an abolishment of the UNFCCC, but rather for the necessity of complementary processes. The most contentious issues in the negotiation arena are currently the division of emission reduction burdens. Here, more intense bilateral or small-n negotiations need to take place. A positive example in this respect is the financial consent on REDD payments by Norway, which gave Brazil enough planning reliability to propose ambitious (yet voluntary) targets in Copenhagen. Forums like the G20 and the Major Economies Meetings may likewise provide a more focused setting for discussing mitigation in a smaller group of key emitters, without being restricted by the shortcomings and slow dynamics of the UN system. Small-n negotiations can provide important impetus and instill new dynamics into the UNFCCC process at large, provided they do so in a transparent and inclusive manner - this was clearly not the case for the CA which has hammered out behind closed doors and then presented to the rest of the 192 Parties as a *fait accompli*. The key objective for Parties should be to use these other negotiation venues to lock the advances made by the CA (on finance, but also on monitoring and reporting of nationally appropriate mitigation actions) into the current UN negotiations [27].

### Finding consensus on equity, fairness and effectiveness

After the Copenhagen experience, rebuilding trust between developing and developed nations is of utmost priority for getting the climate negotiations back on track. Developed countries acting on their promises on finance through the swift operationalization and replenishment of the promised funding mechanisms under the CA using transparent, democratic, flexible and robust rules, would provide a major step forward in this regard. At the same time the CA should be brought back as purely political agreement into the AWG process in order to respect the work of all parties during the last years. Otherwise, parallel agreements might emerge, which would confuse the negotiation process.

Avoiding 'dangerous' climate change must remain the overarching target for the UNFCCC process. Thus, there is a need to re-establish the science basis of climate policy. Currently, proposed medium-term emission targets until 2020 fall far short of the emissions abatement quantities necessary for keeping the 2°C objective within reach. We suggest re-negotiating emission reduction pledges based on a global carbon budget approach [3] in order to achieve coherence between politics and science. This would require to limit global emissions to 44 GtCO_2_e by 2020 under the 2°C guardrail [28]. According to the budget approach the remaining allowable carbon emissions would be distributed globally based on the principle of equal emission rights per capita. This could prevent the mentioned vicious circle of lengthy bargaining on individual country mitigation targets, while providing an acceptable approach of equity and fairness to most Parties [29].

The severe restrictions on the global carbon budget also imply that loopholes such as the assigned amount unit (AAU) surplus takeover from the first commitment period of the Kyoto Protocol need to be closed [30] There are several solutions to this challenge such as restricting the surplus towards domestic compliance in the coming commitment periods, putting a discount on the traded surplus credits, or buying those credits off the compliance market. Obviously, the advantages for the environment and the market have to be balanced against the political acceptability of the solution and the resulting consequences for success on further rounds of negotiation. Except for buying the surpluses, the other solutions bear a tendency of punishment to the surplus holder. International government-financed investment reserves could purchase surplus credits and partially re-sell them to smoothen short-term market imbalances and thus close the mentioned loophole (see e.g. [31]).

### Managing topical complexity

In order to speed up the progress on highly technical issues, such as methodological questions on REDD+ and the details of a global architecture for climate financing, a permanent expert body operating under the Subsidiary Body for Scientific and Technological Advice (SBSTA), could provide for additional negotiation space (see e.g. [31]). Different from several topics where progress is largely determined by diplomatic bargaining during the UNFCCC meetings, these technical questions often overburden the delegates in the limited time available. The permanent expert body could consist of thematic experts and negotiators representing certain country groups. Its role would be to prepare COP decisions and to coordinate the different country proposals on methodological and technical aspects, which need in-depth analysis. Such a body as well as small-n working groups linked to the UNFCCC negotiations could quickly react to new emerging issues and provide the necessary flexibility to adapt to the dynamic nature of the negotiation context.

### Constructing a sound legal basis and protocol architecture

A single legal framework for a post-2012 regime has been one of the negotiating priorities of the EU up until Copenhagen. Yet given the vehement opposition of the BASIC countries and the US to such an approach, it is high time for the EU to rethink its position on this issue. A two-protocol approach may well be the only achievable compromise on legal form. Whereas binding emission reduction targets would continue to apply to industrialized countries under an updated Kyoto Protocol, a second protocol could enshrine no-lose emission reduction targets for economies in transition and the US. Alternatively, the second protocol could include dual intensity targets that require countries to meet a "compliance" target, but allow them to sell credits for emissions reductions above a higher "selling" target [32]. Although both approaches would endorse a substantial degree of inequality among players, they might have the potential to overcome the stumbling block in the negotiations. Asheim (2006) also supports the idea of two separate climate agreements being more effective than one single global agreement from a game-theoretic perspective. As mentioned before, the global budget approach offers an alternative to individually negotiated targets due to equal per-capita emission allowances, which could nevertheless be defined as national targets. As such, they do not only provide a fallback option to the above-mentioned strategies, but rather the basis for target determination in line with the two-protocol approach.

### Enabling climate financing

One tangible outcome of the CA is the immediate establishment of a High Level Panel to study the contribution of the potential sources of revenue, including alternative sources of finance [20]. When submitting its final recommendation immediately before the Cancun COP16 meeting, providing feasible solutions for finance transfer conditions, burden sharing and fast-start operationalization should be high agenda items for this panel. The success on these items will have important repercussions on the trust-building process between developed and developing countries, which is a crucial pre-requisite for advancing in a global climate deal [33]. Although the magnitude of funding will need to be raised considerably to cover the costs of climate change, the urgency of this topic is probably relatively low. In the coming years it will rather be a major challenge to guarantee the absorption capacity for the provided funds given the still tentative developments of many National Adaptation Programmes of Action (NAPA), challenging governance situations and capacity building needs to ensure international measurement, reporting and verification requirements endorsed under the CA. This challenge should not be overlooked, especially since funding for adaptation under the CA will be prioritized for states with rather low absorptive capacity, such as least developed countries and small island developing states.

### Rethinking short-term incentive strategies

Economics and policy need to work closer together to overcome the fundamental collective action dilemma of international climate policy. We argue that recent advances in game theory and innovation economics point towards first-mover advantages for leaders in the climate arena. Dynamic game theory models of coalition formation (perceiving international climate negotiations as a sequential game framework) show that the prospect of future renegotiations and the use of transfer schemes (assume surplus sharing of the gains from cooperation between coalition members) could result in low cost abatement options and incentives to join an international climate agreement at an early stage [34]. To assess the possible benefits of cooperation it is however crucial to increase the transparency and predictability in the negotiations. Conditional emission reduction targets, such as those proposed by the EU could be a feasible way forward, but only if in combination with unconditional lower target bounds.

Furthermore, the often perceived economic disadvantages from ambitious emission reduction pledges mostly rely on static and short-sighted economic considerations. However, if taking into account perspectives of innovation economics, cooperating on stringent climate policy (or even moving forward unilaterally) can yield competitive technical and economic advantages in the unavoidable path to low-carbon economies. Ambitious climate regulations and investments in low carbon frontier technologies will unfold learning processes on research, development and finally diffusion in highly competitive international markets. These learning processes often show path-dependency since a success-breeds-success mechanism [35] provides a lasting lead of the early innovators [36]. Even if they lag behind in efficiency early on, the learning dynamics will ultimately lower the costs of the necessary economic transitions and transform them into an efficiency lead [37]. Hence, in the mid-term freeriding by not investing in emission reduction technologies is counter-productive, since it delays investments in technology to gain first-mover advantages for future markets such as renewable energy technologies [38].

Additionally, progressive countries in the international climate negotiations will be able to take the lead in shaping their future rules and mechanisms. This is in stark contrast to the current negotiation attitude, where the slowest and least constructive one is considered to win. While this assumption might be valid in a short-term perspective, the slowest one looses opportunity and power to decide when change is unavoidable - both economically and politically.

Leadership, credibility and communication are critical for a successful management of change [39]. Independently to any legal commitment, several US states and many cities have set up emission ceilings and emission trading systems. Economies in transition such as China, India and Brazil have passed national laws to reduce emissions, have established national agencies to examine the matter in more detail, and are raising public awareness on climate change. These bottom-up initiatives need to be more strongly visible in international negotiations to demonstrate that mitigation is feasible and attractive. Inspired by the "Fossil of the Day" award by non-governmental organizations which rewards the key laggards and footdraggers during the COPs, the UNFCCC Secretariat or KP parties could turn this concept around and award prizes to local forerunner actions, bottom-up networks or even countries that show success in mitigation and help improve the acceptance of mitigation actions.

## Conclusions

The urgency to increase efforts in emissions abatement and adaptation financing prohibit a further business-as-usual in climate policy. The climate conference in Copenhagen did not succeed in removing the stumbling blocks on the road towards a fair, ambitious and binding Post-2012 agreement. In this paper we analyzed these challenges in order to discuss - without aiming to be exhaustive - alternative solutions to regain momentum in international climate politics.

Timely, fair and equitable solutions require more flexibility in institutions and strategies, a sober analysis of the scientific and political requirements, while simultaneously demanding more pragmatism and a culture of cooperation. We hope to have shown that there are several options and reasons to step off the beaten path in international climate policy in order to tackle the mentioned challenges. Consequently, the complex dynamics of negotiations will require all available efforts to regain momentum for a Post-2012 agreement in the rapidly closing window of opportunity to avoid 'dangerous' climate change.

## Competing interests

The authors declare that they have no competing interests.

## Authors' contributions

MH conceived this study, contributed to all sections and coordinated the main writing process. AF designed the logical sequence of the study and contributed significantly to the discussion section. CH provided valuable input for the policy analysis and discussion sections. UC contributed significantly to aspects of innovation economics in the discussion section. All authors read and approved the final manuscript.
